# Bottom Electrode Effects on Piezoelectricity of Pb(Zr_0.52_,Ti_0.48_)O_3_ Thin Film in Flexible Sensor Applications

**DOI:** 10.3390/ma16237470

**Published:** 2023-12-01

**Authors:** Yanling Yuan, Ling Gao, Naixin Li, Jiuning Gao, Yu Yan, Yiming Zhao, Zongqiang Ren, Hongxin Gong, Yunfei Zhang, Yongbin Liu, Ming Wu, Lisheng Zhong

**Affiliations:** 1 State Grid Jibei Electric Power Co., Ltd., Tangshan Power Supply Company, Tangshan 063000, China; 2 Big Data Center of State Grid Corporation of China, Beijing 100031, China; 3 State Key Laboratory of Electrical Insulation and Power Equipment, Xi’an Jiaotong University, Xi’an 710049, China

**Keywords:** PZT, piezoelectric effect, flexible sensor, ferroelectric thin film

## Abstract

Piezoelectric thin films grown on a mechanical, flexible mica substrate have gained significant attention for their ability to convert mechanical deformation into electrical energy though a curved surface. To extract the generated charge from the PZT thin films, bottom electrodes are typically grown on mica substrates. However, this bottom electrode also serves as a buffering layer for the growth of PZT films, and its impact on the piezoelectric properties of PZT thin films remains understudied. In this work, the effect of Pt and LaNiO_3_ bottom electrodes on the piezoelectric effect of a Pb(Zr_0.52_,Ti_0.48_)O_3_ thin film was investigated. It was observed that the PZT thin films on LNO/Mica substrate possessed weaker stress, stronger (100) preferred orientation, and higher remanent polarization, which is beneficial for a higher piezoelectric response theoretically. However, due to insufficient grain growth resulting in more inactive grain boundaries and lattice imperfections, the piezoelectric coefficient of the PZT thin film on LNO/Mica was smaller than that of the PZT thin film on a Pt/Mica substrate. Therefore, it is concluded that, under the current experimental conditions, PZT films grown with Pt as the bottom electrode are better suited for applications in flexible piezoelectric sensor devices. However, when using LNO as the bottom electrode, it is possible to optimize the grain size of PZT films by adjusting the sample preparation process to achieve piezoelectric performance exceeding that of the PZT/Pt/Mica samples.

## 1. Introduction

Piezoelectric materials have gained remarkable attention in the field of materials science and engineering due to their unique ability to convert mechanical deformation into electrical energy and vice versa [1,2]. Among these materials, lead zirconate titanate (PZT) stands out as a prominent candidate due to its outstanding piezoelectric properties and diverse applications, including sensors, actuators, transducers, and energy harvesting devices [3,4,5]. The piezoelectric performance of PZT is particularly crucial in these applications, and researchers have been continually exploring ways to enhance and optimize it.

In recent years, mica substrates have gained attention as potential platforms for growing PZT thin films [6,7,8]. Mica substrates provide a solid foundation for the growth of PZT thin films while offering the crucial advantage of mechanical flexibility. This flexibility allows PZT films to conform to various shapes and surfaces, making them ideal for applications in which conventional rigid substrates may not suffice. The ability of mica to serve as a flexible support for PZT thin films opens up new possibilities for the development of conformal sensors, wearable devices, and adaptive structures [9,10,11,12,13,14].

In the realm of mica-based flexible sensors, noteworthy achievements have been made. For example, researchers have successfully manufactured a flexible piezoelectric acoustic emission sensor by depositing Pb(Zr_0.52_,Ti_0.48_)O_3_ thin films onto mica substrates [9]. This innovative sensor can be securely affixed to the curved surface of a power cable, enabling the detection of ultrasound signals generated by partial discharges. A flexible vibration sensor was created by depositing BaTiO_3_-based ferroelectric thin films on mica substrates, facilitating structural health monitoring in aircraft [10]. Mica-supported PZT sensors have been employed for pinpointing pipeline leaks, showcasing remarkable sensitivity at 424 mV/MPa^−1^, with an impressive linearity coefficient of 0.99 and a rapid response time of just 0.578 s [11]. Furthermore, mica has proven advantageous in the realm of flexible energy-related technologies [12]. A series of flexible capacitive energy storage devices and energy harvesters have been developed by depositing ferroelectric thin films onto mica substrates [13]. A lead-free (Bi,Na)TiO_3_-based thin film deposited on mica substrates has achieved an exceptional energy storage density exceeding 70 J/cm^−3^ [13]. Furthermore, a Sm-doped PMN-PT thin film, fabricated on mica substrates, has demonstrated outstanding energy harvesting capabilities, boasting an output voltage of 6 V and a current density of 150 μA/cm^−2^ [14]. The advancement of mica-based flexible electronic devices, including energy storage capacitors, energy harvesters, and sensors, has significantly expanded the horizons for Internet of Things technology.

However, to fully harness the potential of mica substrates for piezoelectric applications, careful consideration of the bottom electrode (for conductivity and buffering) is essential. The choice of bottom electrode materials can significantly affect the nucleation, growth, and overall piezoelectric properties of PZT thin films. It plays a pivotal role in controlling the domain structure, polarization orientation, and ultimately, the piezoelectric response of the films. Ruan et al. found that ferroelectric films using LaNiO_3_ as a buffer layer have a much lower surface roughness, a preferred (100)-orientation growth, and better ferroelectric properties [15]. Liu et al. found that if a layer of LNO is added as a buffer layer on a Pt/Ti/SiO_2_/Si substrate, the dielectric constant of the Pb_0.82_La_0.08_Sr_0.1_Ti_0.98_O_3_ (PLST) film can be significantly increased, thereby achieving a significant improvement in the dielectric tuning performance of PLST films [16].

Pt and LaNiO_3_ are widely used as bottom electrodes for PZT thin-film deposition, due to their good conductivity, chemical stability, good adhesion to different kinds of substrate and compatibility with PZT materials. However, there are significant differences between Pt and LaNiO_3_. Firstly, Pt exhibits higher electrical conductivity, which contributes to more efficient charge transfer. Secondly, LaNiO_3_ shares the same perovskite structure with PZT, facilitating the preferred orientation growth of PZT crystals. Additionally, the surface energies of Pt and LaNiO_3_ may differ, potentially leading to variations in the behavior of PZT films during growth. These factors collectively influence the piezoelectric performance of PZT films with Pt and LaNiO_3_ as bottom electrodes, and these performance differences have not been systematically investigated in previous research, especially in cases involving mica substrates.

This study systematically investigates the morphology, structure, ferroelectric properties, dielectric properties, and piezoelectric performance of PZT films when using Pt and LaNiO_3_ as bottom electrodes on mica substrates and compares them with PZT films deposited on Pt/SiO_2_/Si substrates, because Pt/SiO_2_/Si substrates are widely used to prepare ferroelectric thin films [17]. The results indicate that the grain size of PZT films grown on Pt/Mica substrates (abbreviated as Pt/Mica sub) is comparable to that of PZT films grown on Pt/SiO_2_/Si substrates (abbreviated as Pt/Si sub), while the grain size of PZT films grown on LaNiO_3_/Mica substrates (abbreviated as LNO/Mica sub) is slightly smaller. The use of an LNO/Mica substrate facilitates a strong (100) preferred orientation in PZT films. The PZT thin films on Pt/Si and LNO/Mica substrates have similar ferroelectric properties while the remanent polarization of PZT thin film on Pt/Mica substrate is reduced. The experimental results show that, compared to Pt/Si and LNO/Mica substrates, PZT films grown on Pt/Mica substrates exhibit the highest piezoelectric response. Therefore, under the present fabrication method, Pt/Mica substrates are more suitable for PZT film growth to fabricate high-performance, flexible piezoelectric thin-film devices.

## 2. Experiments

The ferroelectric thin films were produced via the sol–gel method [18]. Lead acetate trihydrate (Pb(CH_3_COO)_2_·3H_2_O) (99.998%, Shanghai Aladdin Biochemical Technology Co., Ltd., Shanghai, China), zirconium-n-propoxide (Zr(OC_3_H_7_)_4_) (70%, Shanghai Macklin Biochemical Co., Ltd., Shanghai, China), and titanium isopropylate (Ti(OCH(CH_3_)_2_)_4_) (97%, Meryer (Shanghai) Biochemical Technology Co., Ltd., Shanghai, China) were used as raw materials to synthesize precursor solutions with a composition of Pb(Zr_0.52_,Ti_0.48_)O_3_. Acetic acid (≥99.7%, Alfa Aesar (China) Chemical Co., Ltd., Shanghai, China) and 2-methoxyethanol (99%, Alfa Aesar (China) Chemical Co., Ltd., Shanghai, China), mixed at a 1:4 ratio and used as cosolvents, were stirred for 60 min. To prevent the formation of the pyrochlore phase in the films during crystallization and compensate for lead loss, a 10% excess of lead acetate trihydrate was employed. The resulting solution had a final concentration of 0.4 M. After a 24 h aging period, PZT thin films were deposited on Pt/Si, LNO/Mica, and Pt/Mica substrates using a multiple-step spin-coating process. Each film underwent spin coating at 3500 rpm for 30 s using a spin coater. Subsequently, the wet film underwent a two-minute bake at 350 °C to remove the solvent and at 500 °C to decompose organic matter. It was then annealed at 650 °C for perovskite phase formation. For the up-growing PZT films, the spin coating and heat treatment processes were iterated 25 times. The Pt/Si substrates were commercially available. The bottom electrode LaNiO_3_ was fabricated via the sol–gel method [19], while the Pt bottom electrode was deposited using pulse laser deposition.

To investigate the ferroelectric and electric properties of the films, Pt top electrodes with an area of 8.1 × 10^−5^ cm^−2^ were deposited on the films using an ion sputtering coater (Quorum Q 150R Plus, East Sussex, England). The crystallization and phase evolution behavior of the films were analyzed using an X-ray diffractometer (XRD, D8 Advance, Bruker, MA, USA) with a step of 0.02° from 20° to 50°. The surface morphology and cross-section of the PZT thin films were determined using a scanning electron microscope (SEM ZEISS GeminiSEM500, Oberkochen, Germany). The measurements of dielectric constant and loss tangent values were performed by using an LCR meter (Agilent E4980A, Palo Alto, CA, USA). The leakage current density was measured using a ferroelectric tester (Radiant Technologies Precision Multiferroic, Albuquerque, NM, USA). The ferroelectric hysteresis loop of the thin films was measured with two tips at a frequency of 1 kHz using a ferroelectric tester (PolyK Technologies LY20-30300, Philipsburg, PA, USA). The mesoscopic domain switching capability and piezoelectric properties of the films were represented using PFM (Asylum research MFP-3D Origin, Santa Barbara, CA, USA).

## 3. Results and Discussion

**Figure 1:**
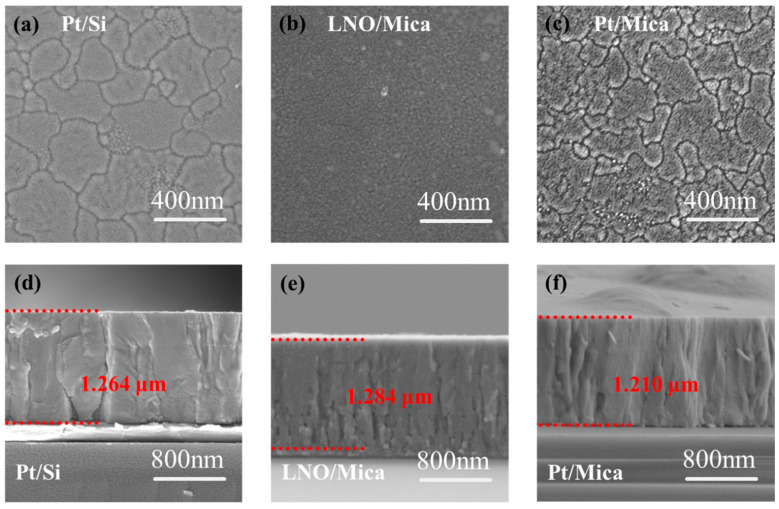
(**a**–**c**) Surface morphology and (**d**–**f**) cross-section morphology of the PZT thin films on Pt/Si substrate, LNO/Mica substrate, and Pt/Mica substrate, respectively.

The surface morphology of the PZT thin films on a Pt/Si substrate, LNO/Mica substrate, and Pt/Mica substrate are shown in Figure 1a–c. It can be seen that the PZT thin films grown on all kinds of substrate show a very compact microstructure, without any visible pore or crack. The grain size of PZT thin films grown on Pt/Si (Figure 1a) and Pt/Mica (Figure 1c) substrates is similar, ranging from tens to hundreds of nanometers. The distribution pattern of grain sizes is also quite consistent. In contrast, PZT thin films grown on an LNO/Mica (Figure 1b) substrate primarily have grain sizes in the tens of nanometers. The grain size can affect the ferroelectric and piezoelectric properties of PZT thin films in two ways [20,21,22]. Smaller grains possess a higher density of ferroelectric domains and require less energy to activate domain wall motion, consequently resulting in a heightened piezoelectric response. Conversely, smaller-sized grains harbor increased inactive areas like grain boundaries and more noticeable lattice imperfections. They exhibit a much stronger depolarization field, which ultimately weakens the overall piezoelectric response compared to larger grains. To obtain piezoelectric thin film materials with excellent piezoelectric responses on flexible mica substrates, further optimization of their grain size is necessary.

The thickness of the PZT thin films was about 1.2 μm for all three kinds of substrates (as shown in Figure 1d–f), which means that the thickness of the thin film is mainly determined by the sol–gel deposition times and rarely affected by the substrates that possess different lattice match and surface energies.

**Figure 2:**
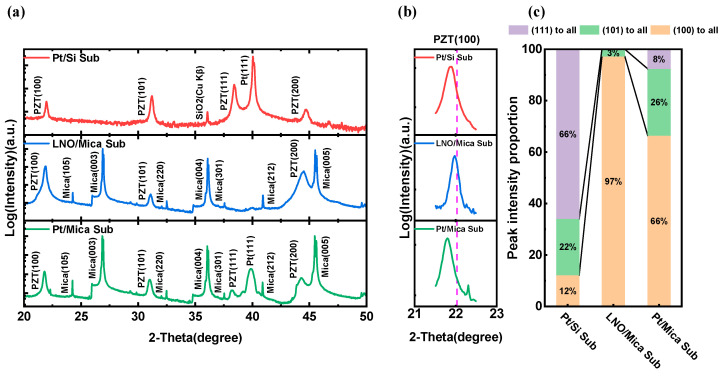
(**a**) XRD curves of the PZT thin films on Pt/Si substrate, LNO/Mica substrate, and Pt/Mica substrate, respectively. (**b**) The enlarged (100) peaks. (**c**) Percentage of (100)-, (101)-, and (111)-oriented PZT thin films on different substrates.

Figure 2a shows the crystallization characteristics of the PZT thin films on Pt/Si substrate, LNO/Mica substrate, and Pt/Mica substrate, respectively. Obvious perovskite crystal structures were observed in the PZT films on all three substrates. The (100) characteristic peaks of the PZT films are enlarged and shown in Figure 2b, in which the dotted lines represent the PZT(100) peak positions of the standard PDF card (PDF#33-0784). The observed slight shift toward lower degrees in the (100) peaks of the PZT thin film suggests the presence of compressive stress within the film. Specifically, the compressive stress appeared notably stronger in the PZT films deposited on Pt/Si and Pt/Mica substrates than those on LNO/Mica substrates.

For the fabricated PZT thin film in this work, its composition is located at the morphotropic phase boundary (MPB) in the PbZrO_3_-PbTiO_3_ phase diagram, which means a mixed phase of tetragonal (T phase) and rhombohedral (R phase) [23,24]. The polarization of Pb(Zr,Ti)O_3_ in the T phase points to the (001) direction, while that in the R phase points to the (111) direction. For (001)/(100)- and (111)-oriented PZT thin films, their polarization can be more easily switched using external stimulation and exhibits a better ferroelectric and piezoelectric response. The orientation preference of PZT films on different substrates could be characterized by the relative intensity of diffraction peaks. Figure 2c further summarizes the percentages of (100)-, (101)-, and (111)-oriented PZT films on the Pt/Si substrate, LNO/Mica substrate, and Pt/Mica substrate, respectively. It can be seen that, for the PZT thin film on Pt/Si substate, the ratio of I_(100)_ + I_(111)_ to I_(100)_ + I_(101)_ + I_(111)_ was 0.78, while that of PZT on LNO/Mica and on Pt/Mica substrate was 0.97 and 0.74, respectively. It was also noticed that the (111) of PZT disappeared when the thin film was deposited on an LNO/Mica substrate. It is evident that the PZT thin film on the LNO/Mica substrate, guided by the LNO layer with the same perovskite structure, exhibits a strong (100) preferred orientation, which may also indicate that the LNO/Mica substrate has the potential to induce greater out-of-plane polarization of PZT films; that is, under the same test conditions, PZT films on an LNO/Mica substrate may exhibit higher remanent polarization.

**Figure 3:**
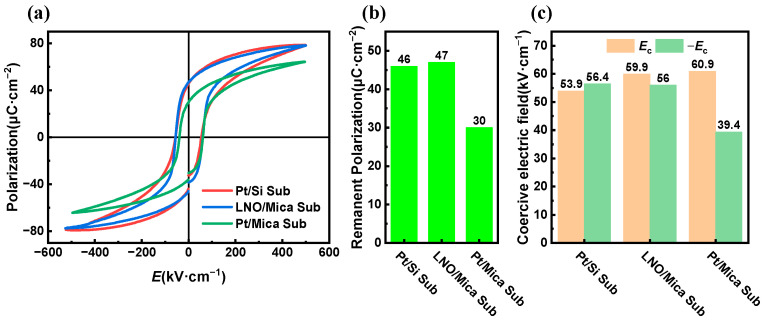
(**a**) Hysteresis loops, (**b**) remnant polarization, and (**c**) coercive field of the PZT thin films on LNO/Mica, Pt/Si, and Pt/Mica substrates.

The electric-field-polarization curves of the PZT thin films shown in Figure 3a exhibit typical hysteresis characteristic [25], indicating the ferroelectric nature of the fabricated PZT thin films on each type of substrate. The remnant polarization (polarization as electric field equal to 0 kV/cm) of the PZT thin film on LNO/Mica and Pt/Si substrate was 47 μC/cm^2^ and 46 μC/cm^2^, respectively, while that of PZT thin films on Pt/Mica substrate was much smaller, which was 30 μC/cm^2^ (as shown in Figure 3b). The remnant polarization of PZT films is jointly influenced by factors such as crystal preferred orientation, grain size, and its distribution, as well as the stress state of the film [26,27]. In general, the larger the remnant polarization, the greater the polarization change generated by piezoelectric materials under external stimulation, leading to a more pronounced piezoelectric effect [28]. From this perspective, mica substrates with LNO buffering are more suitable as growth substrates for flexible piezoelectric thin films.

The PZT thin films deposited on both LNO/Mica and Pt/Si substrates exhibited similar coercive field strengths under positive and negative electric fields, both falling within the range of 55–60 kV/cm, showing good symmetry (as shown in Figure 3c). However, for PZT films on Pt/Mica substrates, the coercive field under a negative electric field was 39.4 kV/cm, significantly smaller than the 60.9 kV/cm observed under a positive electric field. This may be induced by the formation of a dead layer between the PZT film and the Pt substrate. The reversely polarized ferroelectric domain on the interface dead layer is more difficult to nucleate, which leads to a difference in the coercive electric field under positive and negative voltages. The asymmetry of the coercive voltage also leads to different piezoelectric responses under positive and negative voltages, as discussed later. This result suggests that, for PZT films on Pt/Mica substrates, it is easier to be poled using negative voltage.

**Figure 4:**
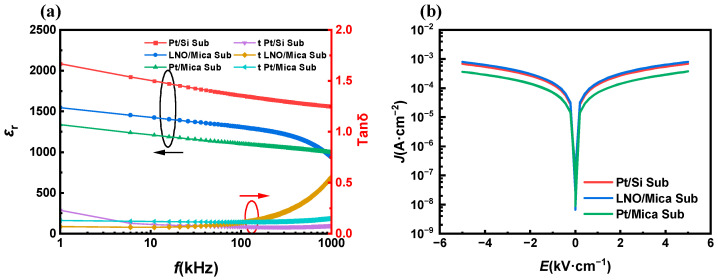
(**a**) Dielectric properties and (**b**) leakage current of the PZT thin films on LNO/Mica, Pt/Si, and Pt/Mica substrates.

The relative dielectric properties of the PZT thin films were measured in the frequency range from 1 kHz to 1 MHz, and the results are shown in Figure 4a. It is seen that the relative dielectric constant of the PZT thin film fell in the range from 1000 to 2000, while that of the PZT thin film on a Pt/Mica substrate was lower than for the other two substrates. The decrease in permittivity and increase in loss observed in PZT thin films on LNO/Mica substrates above 200 kHz are associated with film inhomogeneities. These inhomogeneities alter the distribution of local depolarization fields, stress distribution within the film, as well as the quantity, depth, and density of charge traps. These result in a significant difference in dielectric response at high frequencies compared to low-frequency conditions. In the design of piezoelectric sensor devices, minimizing the relative dielectric constant of the piezoelectric material is preferred. A smaller dielectric constant leads to reduced sensor capacitance. Electrical signals produced by piezoelectric sensors in response to external stimuli typically require amplification through signal amplification circuits before being read by data acquisition equipment. Common amplification methods include voltage amplifiers and charge amplifiers. Voltage amplifiers primarily enhance voltage signals from the piezoelectric sensor. The voltage signal output by the sensor is inversely proportional to its capacitance for a given quantity of charge, resulting in higher sensitivity when the dielectric constant of the piezoelectric material is smaller. Even when charge amplifiers are employed in the backend circuitry, their upper cutoff frequency is likewise inversely proportional to the sensor’s capacitance. If the sensor’s capacitance is excessively high, the piezoelectric sensing system will be limited to low-frequency operation.

Figure 4b shows the leakage current of the PZT thin films. It was observed that the PZT thin film on the Pt/Mica substrate had the lowest leakage current, which is beneficial for the poling process of the PZT thin film, as a higher leakage current always results in electrical breakdown. Therefore, from the perspective of dielectric properties and leakage current, the Pt/Mica substrate is more suitable for the fabrication of flexible piezoelectric sensors.

**Figure 5:**
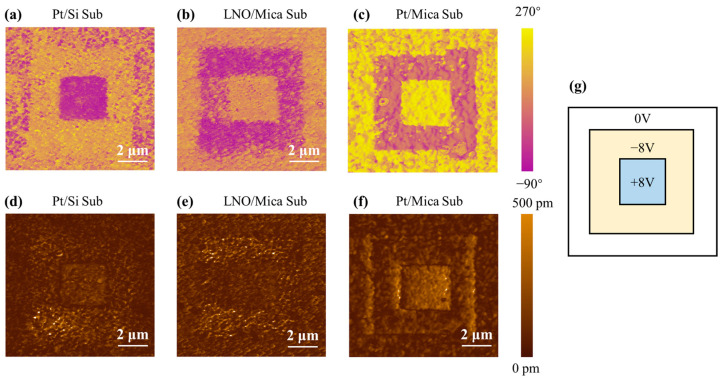
(**a**–**c**) Phase and (**d**–**f**) amplitude images of the PZT thin films. (**g**) Schematic of the scanning voltage.

The piezoelectric response of the PZT thin films was characterized using a piezoelectric force microscope (PFM). The phase images (Figure 5a–c) and amplitude images (Figure 5d–f) revealed the polarization direction and piezoelectric effect of the PZT thin films, respectively. A scanned square with a length of 10 μm was divided into three regions, that is, the initial region without switching located on the outer side, the region switched using a negative voltage of −8 V located at the center, and the regions switched using a positive voltage of 8 V on the inner side, as the schematic shows in Figure 5g.

Figure 5a–c show the phase images of the PZT thin films on the Pt/Si, LNO/Mica, and Pt/Mica substrates, respectively. The distribution of purple and bright yellow in the figure indicates that the polarization directions in this area were opposite. In the initial state of the regions (outer side), phase maps of the PZT films on Pt/Si substrates displayed a uniform distribution, alternating between purple and bright yellow. This indicates that the polarization directions in these areas were randomly distributed (see Figure 5a) [29]. Conversely, in the case of PZT films grown on LNO/Mica and Pt/Mica substrates, the color in the outer regions corresponded to the inner regions polarized in the positive direction. This implies that the PZT films grown on LNO/Mica and Pt/Mica substrates were self-polarized in a downward direction. In the central and inner regions of the PZT thin films, distinct color variations were observed upon applying −8 V and 8 V voltage reversals, signifying differing polarization orientations (Figure 5a–c). This suggests that the polarization of the PZT film can be readily switched by an 8V voltage, irrespective of the substrate type.

In the amplitude images (as shown in Figure 5d–f), the bright yellow color indicates a more active piezoelectric response induced by the AC signal from the PFM scanning tip. At the positions corresponding to the scanning voltage boundaries in Figure 5g, some narrow black rectangles appeared in Figure 5d–f. These were domain walls formed after the polarization switch. It is well known that domain wall regions do not exhibit piezoelectric activity. For the PZT thin films on Pt/Si and Pt/Mica substrates, the scanning-induced domain wall was more distinct than that of PZT thin films on the LNO/Mica substrate. Overall, for the PZT thin films on Pt/Mica substrates, the regions polarized with +8 V voltage exhibited a uniform and strong piezoelectric response. However, on LNO/Mica substrates, the regions polarized with −8 V voltage showed a stronger piezoelectric response, although their distribution was not as uniform.

**Figure 6:**
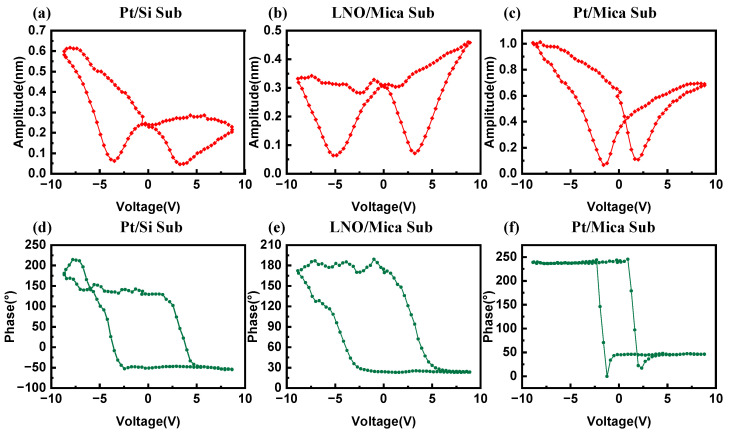
(**a**–**c**) Voltage–amplitude and (**d**–**f**) voltage–phase curves of the PZT thin films.

The in-depth assessment of the piezoelectric response of the PZT thin films involved measuring the local piezoelectric responses, including voltage–amplitude and voltage–phase curves [30], as depicted in Figure 6. Remarkable asymmetry was exhibited in the amplitude curves of PZT films on different types of substrates, likely originating from interface effects between the substrates and the PZT thin films. Furthermore, when subjected to a voltage stimulus of −8 V, the PZT thin film on the Pt/Mica substrate demonstrated a remarkable amplitude response of 1 nm, significantly surpassing that of the other two substrates, indicating the highest piezoelectric response among them. At the same time, its phase curve disclosed an exceptionally abrupt polarization reversal when the applied voltage exceeded the coercive field. This suggests that, considering the current fabrication conditions, mica substrates buffered with Pt as both the bottom electrode and the buffer layer are the preferred option for manufacturing flexible piezoelectric thin films.

## 4. Conclusions

In this work, Pt and LaNiO_3_ were deposited (by pulse laser deposition and by sol–gel method, respectively) on a mica substrate as the bottom electrode and the buffering layer for PZT thin film growth. The effect of Pt and LaNiO_3_ on the piezoelectric properties of the sol–gel fabricated PZT thin film was investigated in detail. Experimental results showed that the PZT thin film on Pt/Mica possessed a larger piezoelectric coefficient, a characteristic that is obviously advantageous for its application in piezoelectric thin-film sensor devices. The PZT thin film on the LNO/Mica substrate exhibited lower stress, stronger (100) preferred orientation, and greater remnant polarization compared with those grown on Pt/Mica substrates, which theoretically favors the superior piezoelectric performance of PZT (LNO/Mica) films. The disparity in these outcomes may be attributed to the smaller grain size of the PZT films on LNO/Mica substrates. The next step holds the potential to optimize the grain size by adjusting the fabrication process for the PZT/LNO/Mica samples, aiming to achieve superior piezoelectric effects surpassing those of PZT/Pt/Mica.

## Data Availability

The data presented in this study are available on request from the corresponding author.
